# Efficacy of Definitive Radiotherapy for Patients with Clinical Stage IIIB or IIIC Lung Adenocarcinoma and Epidermal Growth Factor Receptor (EGFR) Mutations Treated Using First- or Second-Generation EGFR Tyrosine Kinase Inhibitors

**DOI:** 10.1155/2024/8889536

**Published:** 2024-03-05

**Authors:** Chih-Yen Tu, Te-Chun Hsia, Ying-Chun Lin, Ji-An Liang, Chia-Chin Li, Chun-Ru Chien

**Affiliations:** ^1^Division of Pulmonary and Critical Care Medicine, Department of Internal Medicine, China Medical University Hospital, Taichung, Taiwan; ^2^School of Medicine, College of Medicine, China Medical University, Taichung, Taiwan; ^3^Health Science and Industry, College of Health Care, China Medical University, Taichung, Taiwan; ^4^Department of Radiation Oncology, China Medical University Hospital, Taichung, Taiwan

## Abstract

**Background:**

The effectiveness of definitive radiotherapy (RT) for patients with clinical stage IIIB or IIIC lung adenocarcinoma and epidermal growth factor receptor (EGFR) mutations who received first- or second-generation EGFR tyrosine kinase inhibitors (TKIs) is unclear.

**Methods:**

Taiwan Cancer Registry data were used in this retrospective cohort study to identify adult patients diagnosed with EGFR-mutated stage IIIB or IIIC lung adenocarcinoma between 2011 and 2020. Patients treated with first- or second-generation EGFR TKIs were classified into RT and non-RT groups. Propensity score (PS) weighting was applied to balance covariates between groups. The primary outcome was overall survival (OS), and the incidence of lung cancer mortality (ILCM) was considered as a supplementary outcome. Additional supplementary analyses were conducted to assess the robustness of the findings.

**Results:**

Among 270 eligible patients, 41 received RT and 229 did not. After a median follow-up of 46 months, PS-weighted analysis showed the PS-weighted hazard ratio of death for the RT group compared to the non-RT group was 0.94 (95% CI: 0.61–1.45, *p* = 0.78). ILCM rates did not differ significantly between the two groups. Supplementary analyses yielded consistent results.

**Conclusion:**

The addition of definitive RT to first- or second-generation EGFR TKI treatment does not significantly improve OS of patients with EGFR-mutated stage IIIB or IIIC lung adenocarcinoma. NCT03521154NCT05167851.

## 1. Introduction

Lung cancer is a leading cause of cancer-related mortality worldwide, including in Taiwan [1, 2]. Non-small-cell lung cancer (NSCLC), particularly adenocarcinoma, represents the majority of cases of lung cancer in Taiwan [2]. The treatment approach for advanced-stage NSCLC depends on biomarker profiling [3]. For patients with epidermal growth factor receptor (EGFR) mutations, specifically the common mutations, exon 19 deletion or exon 21 L858R, the category-1 recommendation of the National Comprehensive Cancer Network (NCCN) guidelines is the use of EGFR tyrosine kinase inhibitors (TKIs) [3]. Currently, approved EGFR TKIs include gefitinib, erlotinib, afatinib, dacomitinib, and osimertinib [3].

Nevertheless, the role of EGFR TKIs for the treatment of locally advanced stage NSCLC remains uncertain. For patients with stage IIIB to IIIC disease, who are not eligible for surgical resection, the NCCN suggests definitive concurrent chemoradiotherapy (dCCRT) [3]; however, this approach has been the subject of debate in the literature [4], and our previous study reported comparable outcomes between patients treated with EGFR TKIs alone and those treated with dCCRT [5].

Currently available EGFR TKIs can be classified as first (e.g., gefitinib or erlotinib), second (e.g., afatinib or dacomitinib), and third (e.g., osimertinib) generation [6]. A comprehensive National Health Insurance (NHI) system has been implemented in Taiwan since 1995. First- or second-generation EGFR TKIs have been approved by the NHI as a first-line treatment for patients with at least stage IIIB lung adenocarcinoma and EGFR mutations [7]. Consequently, many patients with lung adenocarcinoma and EGFR mutations in Taiwan have been treated with first- or second-generation EGFR TKIs as their first-line treatment, rather than the guideline-recommended dCCRT [3, 5].

Drug resistance is a common issue in patients treated with EGFR TKIs [6]. A randomized controlled trial (RCT) including patients with oligometastatic lung adenocarcinoma and EGFR mutations receiving first generation EGFR TKIs, SINDAS, reported that the addition of radiotherapy (RT) significantly improved overall survival (OS) [8]. Another RCT for patients with unresectable stage III NSCLC with EGFR mutations showed that erlotinib plus RT significantly improved progression-free survival relative to dCCRT [9]. A search conducted on PubMed using the terms, “((non-small-cell lung) OR (lung adenocarcinoma)) AND ((locally advanced) OR (stage III)) AND (EGFR mutation) AND ((radiation) OR (radiotherapy)) AND (gefitinib OR erlotinib OR afatinib OR dacomitinib OR osimertinib OR (tyrosine kinase inhibitor^*∗*^)),” in June 2023 yielded no relevant RCTs, only the observational KINDLE study [10, 11]. Given the aforementioned controversy and the limited literature available on this topic, we designed this retrospective cohort study to investigate the efficacy of definitive RT for patients with clinical stage IIIB or IIIC lung adenocarcinoma and EGFR mutations treated with first- or second-generation EGFR TKIs using a population-based approach.

## 2. Materials and Methods

### 2.1. Data Sources

This retrospective cohort study used deidentified databases obtained from the Health and Welfare Data Science Center, Ministry of Health and Welfare, which included comprehensive information from the Taiwan Cancer Registry (TCR), the death registry, and reimbursement data for the entire population of Taiwan, provided by the Bureau of NHI. The study was approved by the Central Regional Research Ethics Committee of China Medical University, Taichung, Taiwan (CRREC-108-080 (CR-3)).

### 2.2. Study Design, Population, and Intervention

Adult patients aged 18–75 years [12] diagnosed with EGFR-mutated stage IIIB or IIIC lung adenocarcinoma [13] were identified according to the American Joint Committee on Cancer 7th or 8th edition clinical staging criteria, [14, 15] between 2011 and 2020. These patients had a good performance status (PS) (Eastern Cooperative Oncology Group (ECOG) PS 0–2) and were treated with first or second-generation EGFR TKIs [6], as indicated in the TCR, because the NHI reimbursed gefitinib, erlotinib, afatinib, and dacomitinib for patients with lung adenocarcinoma during the study period (2011–2020), but not other EGFR TKIs. Patients who underwent surgery, received other systemic therapies (including chemotherapy), had multiple treatment records, or had prior cancers were excluded to ensure data quality.

**Figure 1:**
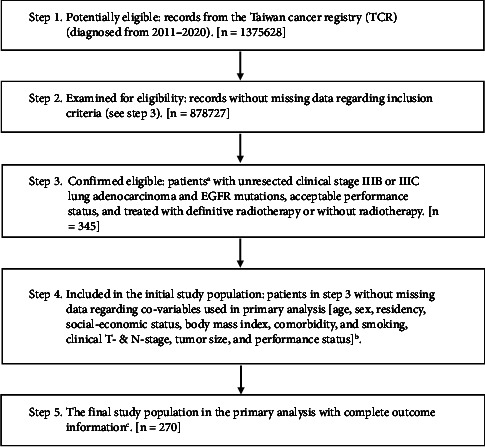
STROBE study flowchart and the number of individuals at each stage of the study. ^a^Only those treated (class 1-2) and with a single record were included, to ensure data consistency. ^b^The exact numbers are not reported because of a health and welfare data science center database center policy to avoid numbers ≤2 in single cells. ^c^Without missing information in the TCR and death registry regarding survival status.

Patients were classified into an RT group (treated with definitive RT) and a non-RT group (without RT). Patients included in the RT group received external beam RT with equivalent doses in 2-Gy per fractions, assuming an alpha/beta ratio of 10 (EQD2 (10)), [16] within the range of 31.25–70 Gy, as per the literature [3, 8]. The explanatory variable of interest was the receipt of RT (RT group vs. non-RT group), and the primary outcome was OS. The incidence of lung cancer mortality (ILCM) was considered as a supplementary outcome. Information on OS and ILCM was obtained through linkage with the TCR or death registry records. The index date was defined as the date of diagnosis, and OS/ILCM was calculated from the index date to the date of death or December 31, 2021 (the censoring date of the death registry). A study flowchart is shown in Figure 1, as suggested by the STROBE guideline [17].

### 2.3. Covariates

To account for potential nonrandomized treatment selection, the following covariates were collected, based on recent relevant studies and our clinical research experiences: [5, 8–10, 18] patient demographics (age, sex, residency region, and socioeconomic status (SES)), patient characteristics (body mass index (BMI), comorbidity, smoking, and performance status), and disease characteristics (clinical T- and N-stage, tumor size).

Patient residency region was classified as northern or non-northern Taiwan, based on previous studies reporting geometric differences in disease characteristics [19]. Sex was classified as male or female. SES was classified as higher (income greater than the minimum wage) or not. Comorbidity was determined using the modified Charlson comorbidity index score and classified as ≥1 or <1 [20]. Smoking status was classified as yes or no. Clinical T-stage was classified as (1-2) or (3-4), while the clinical N-stage was classified as (0–2) or 3. ECOG PS was classified as (0-1) or 2. Age (years), BMI (kg/m^2^), and tumor size (mm) were treated as continuous variables.

### 2.4. Statistical Analysis and Supplementary Analyses

In the primary analysis (PA), a propensity score (PS) approach was employed, using a logistic regression model with the aforementioned covariates to balance measured potential confounders [21]. PS weighting (PSW) was used, with the overlap weight as the framework [22–25]. After verifying covariate balance between groups using standardized difference after PSW, [26, 27] the hazard ratio (HR) of death was compared between the RT and non-RT groups [28]. Point estimates were calculated using a Cox proportional hazards model in the weighted sample throughout the entire follow-up period [28, 29]. The 95% confidence interval (95% CI) was estimated using the bootstrap method [30, 31].

To assess the robustness of the findings regarding potential unmeasured confounders, the *E*-value was applied, as suggested in the literature [32, 33]. The incidence of lung cancer mortality between the RT and non-RT groups was evaluated using the competing risk approach in the weighted sample [34]. The International Classification of Diseases, Ninth Revision, Clinical Modification (ICD-9-CM) and ICD-10-CM coding (5080/5081 and J700/J701, respectively [35]) were used to estimate the rate of radiation pulmonary toxicity within 6 months after RT in the RT group.

Supplementary analyses (SA) included four additional analyses, to evaluate the robustness of the findings. In SA-1, an alternative analytical approach, PS matching (PSM), was applied by constructing a 1 : 1 PS-matched cohort (without replacement) within the primary study population. The HR of death was compared using a robust variance estimator [28]. The subdistribution HR was used to assess ILCM with the clustered Fine-Gray model [36]. In SA-2, the RT dose was limited to at least EQD2 (10) 50 Gy [37] for the RT group in the PA, to explore its impact in the RT and non-RT groups. In SA-3, impact was evaluated by limiting patients to those with common mutations (Exon 19 deletion or Exon 21 L858R [3, 9]), which were recorded in the TCR since 2019. In SA-4, patients who received palliative RT, according to TCR records, were excluded in the PA.

Statistical analyses were performed using SAS 9.4 software (SAS Institute, Cary, NC, USA) and R version 4.3.0 (R Development Core Team, R Foundation for Statistical Computing, Vienna, Austria).

## 3. Results

### 3.1. Study Population and Descriptive Data

As shown in Figure 1, our study population consisted of 270 eligible patients with unresected clinical stage IIIB or IIIC lung adenocarcinoma and EGFR mutations who received RT (41 patients) or not (229 patients) between 2011 and 2020. Patient characteristics are described in Table 1. All covariates achieved balance (standardized differences approximately 0) after PS weighting via the overlap weights, although patient residency region was imbalanced before PSW [26].

### 3.2. Primary Analysis

**Figure 2:**
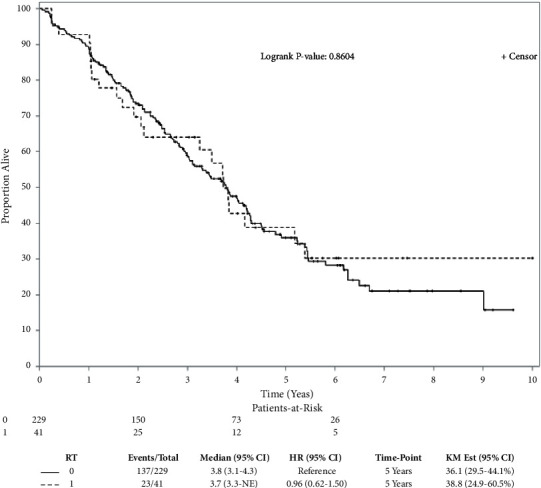
Kaplan–Meier overall survival curve (in years) showing no significant difference between the RT and non-RT groups in the primary analysis before adjustment for covariates. RT, radiotherapy.

**Figure 3:**
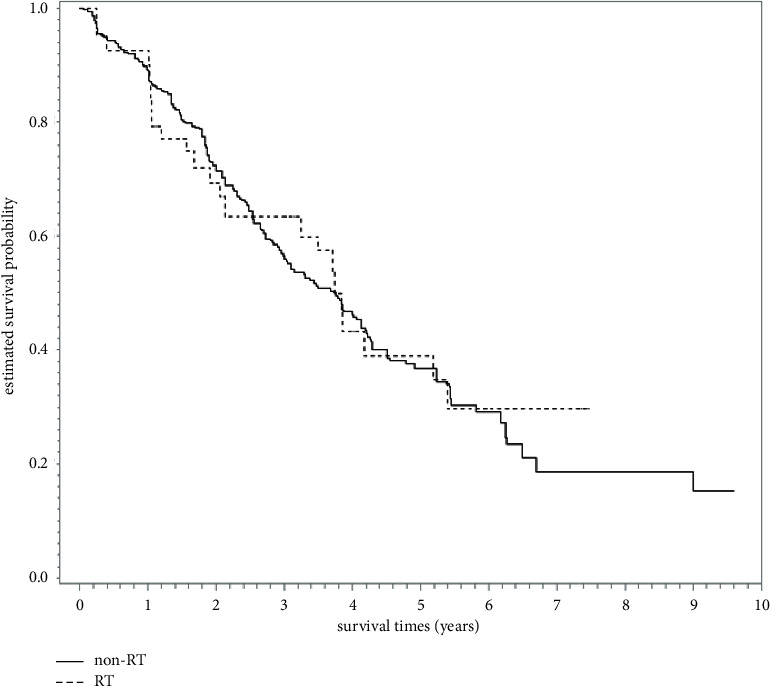
Overall survival curve (in years) showing no significant difference between the RT and non-RT groups in the primary analysis after overlap weights adjustment. RT, radiotherapy.

After a median follow-up of 46 months (range, 12–127 months), 160 deaths were observed (23 and 137 patients in the RT and non-RT groups, respectively). In the unadjusted analysis, the 5-year OS rates were 39% (RT group) and 36% (non-RT group) (log-rank test, *p*=0.86) (Figure 2). The overlap weight-adjusted OS curve is shown in Figure 3. The 5-year PSW-adjusted OS rates were 39% (RT group) and 37% (non-RT group). Relative to the non-RT group, the PSW-adjusted HR of death for the RT group was 0.94 (95% CI = 0.61–1.45, *p*=0.78), which could be explained by an unmeasured confounder associated with the selection of treatment (RT or non-RT) and survival with risk ratios of 1.26 (E-value)-fold each, but not by weaker confounding factors. No significant difference in ILCM was detected between groups (HR = 0.97, *p*=0.92). Of patients in the RT group, 15% [6/41] developed radiation pulmonary toxicity.

### 3.3. Supplementary Analyses

**Figure 4:**
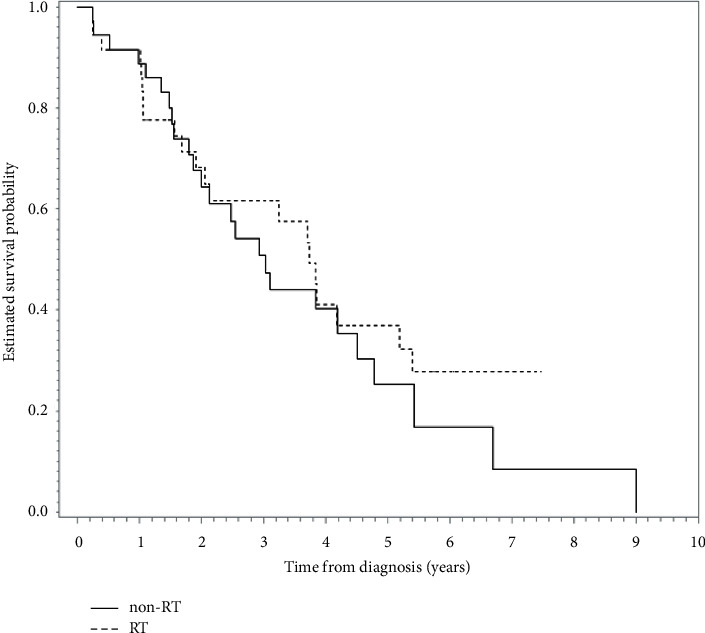
Kaplan–Meier survival curve (in years) showing no significant difference between the RT and non-RT groups in the first supplementary analysis (propensity score-matched analyses). RT, radiotherapy.

The constructed PS-matched subgroup (*n* = 72) used in SA-1 is shown in Table 2; all covariates were balanced after PSM. A Kaplan–Meier OS curve is presented in Figure 4. The 5-year OS rates were 37% (RT group) and 25% (non-RT group), and there was no significant difference between the groups (HR = 0.78, 95% CI = 0.45–1.35, *p*=0.37), with a similar result for comparison of ILCM (HR = 0.76, *p*=0.32).

Among the 264 patients included in SA-2 (Table 3), we found that the PSW-adjusted HR of death was 0.83 (95% CI = 0.49–1.39, *p*=0.47) when the RT group (at least EQD2(10) 50 Gy) was compared to the non-RT group, and the result for ILCM remained similar (HR = 0.85, *p*=0.65).

In SA-3 (Table 4), we found that OS remained similar between the groups (PSW-adjusted HR = 0.89, *p*=0.99) for patients with common mutations (Exon 19 deletion or Exon 21 L858R), and a similar result was observed for ILCM (HR = 0.89, *p*=0.93).

In SA-4 (supplementary material Table S1), we again found no significant difference in OS between the RT group, which excluded those who received palliative RT, and the non-RT group (PSW-adjusted HR = 0.78, *p*=0.32); further, comparison of ILCM generated a similar result (HR = 0.79, *p*=0.51).

## 4. Discussion

In this retrospective cohort study based on a nationwide cancer registry, we investigated the effectiveness of definitive RT for patients with locally advanced stage lung adenocarcinoma and EGFR mutations treated with first or second generation EGFR TKIs. Our findings suggest that the OS of patients with EGFR mutations is similar whether or not RT is added to EGFR TKI treatment. Our results were consistent across different analyses, including of ILCM and SA.

To the best of our knowledge, this is the first population-based study to explore this specific topic. The outcomes of our investigation, which showed no statistically significant effect of RT on OS, align with the findings reported in the KINDLE study, [10, 11] as the 95% confidence intervals overlapped; however, it is important to note that the KINDLE study reported a numerically longer median OS for the RT group (42.6 months) compared to the non-RT group (25.4 months), whereas our study found similar OS values in both groups.

Given the nonrandomized and registry-based nature of our study, our findings should be interpreted with caution. It will be essential to conduct further prospective studies, particularly RCTs, to investigate the effect of RT in patients treated with third generation EGFR TKIs. Although we are not aware of any ongoing RCTs specifically addressing this question, two related trials, namely, the LAURA trial (NCT03521154) and the ABLATE trial (NCT05167851), [38, 39] may offer additional insights in the future. The LAURA trial is designed to examine the role of consolidative osimertinib in locally advanced NSCLC with EGFR mutation treated with dCCRT, while the ABLATE trial is to investigate the role of upfront RT for oligometastatic NSCLC with EGFR mutation treated by lazertinib (another third generation EGFR TKI). Furthermore, gefitinib was the first EGFR TKI approved by NHI for locally advanced NSCLC, followed by erlotinib, afatinib, and dacomitinib; therefore, our results are not applicable to other first or second generation EGFR TKIs, such as icotinib.

Several limitations should be acknowledged in our study. First, the potential for unmeasured confounders always exists in this type of nonrandomized study. To evaluate the robustness of our findings to potential unmeasured confounders, we employed the *E*-value, which provides a measure of the minimum strength of associations that an unmeasured confounder would need to have with both the treatment and the outcome to explain away the observed effect. Second, although EGFR TKIs are indicated for treatment in the context of common mutations (exon 19 deletion and exon 21 L858R), which account for around 85% of patients with EGFR mutations, [40] and are also indicated for some less common mutations (such as EGFR S768I, L861Q, and/or G719X), [3] the potential inclusion of insensitive rare EGFR mutations, such as exon 20 insertions, [3, 41] may limit the clinical implications of our findings; however, the incidence of exon 20 insertions among patients with EGFR mutations in Taiwan is low (3%-4%) [41]. Finally, due to data limitations within the cancer registry, we neither included other endpoints, such as progression-free survival or quality of life, nor investigated the effect of postprogression treatment.

## 5. Conclusion

Our study suggests that the addition of RT to first or second generation EGFR TKI treatment did not significantly impact the OS of patients with clinical stage IIIB or IIIC lung adenocarcinoma and EGFR mutations; however, further research is needed to validate our findings, particularly in the context of third generation EGFR TKIs. RCTs investigating the role of RT in this patient population are eagerly awaited to provide more definitive evidence.

## Figures and Tables

**Table 1:** Characteristics of the patient study population included in the primary analysis.

		RT group (*n* = 41)		Non-RT group (*n* = 229)		Standardized difference^†^	
		Number or mean (SD)^†^	(%)^†^	Number or mean (SD)^†^	(%)^†^	Before PSW	After PSW
Age (years)		63.61 (8.35)		64.60 (8.30)		0.119	≈0

Sex	Female	24	(59)	158	(69)	0.219	≈0
	Male	17	(41)	71	(31)		

Residency	Non-northern	22	(54)	160	(70)	0.338	≈0
	Northern	19	(46)	69	(30)		

Comorbidity	<1	38	(93)	204	(89)	0.125	≈0
	≥1	3	(7)	25	(11)		

BMI (kg/m^2^)		25.07 (5.15)		24.01 (3.71)		0.238	≈0

Social-economic status	Minimum wage or lower	9	(22)	70	(31)	0.197	≈0
	Higher	32	(78)	159	(69)		

Smoking	No	29	(71)	178	(78)	0.161	≈0
	Yes	12	(29)	51	(22)		

Clinical T-stage	T1–T2	17	(41)	95	(41)	0	≈0
	T3–T4	24	(59)	134	(59)		

Clinical N-stage	N0–N2	11	(27)	49	(21)	0	≈0
	N3	30	(73)	180	(79)		

Tumor size (mm)		45.71 (19.23)		42.97 (20.74)		0.137	≈0

ECOG PS	0-1	36	(88)	212	(93)	0.161	≈0
	2	5	(12)	17	(7)		

BMI, body mass index; ECOG PS, eastern cooperative oncology group performance status; PSW, propensity score weighting; RT, radiotherapy; SD, standard deviation. ^†^Rounded.

**Table 2:** Characteristics of patients included in the first supplementary analysis.

		RT group (*n* = 36)		Non-RT group (*n* = 36)		
		Number or mean (SD)^†^	(%)^†^	Number or mean (SD)^†^	(%)^†^	Standardized difference^†^

Age (years)		63.19 (8.56)		63.00 (6.09)		0.243

Sex	Female	20	(56)	21	(58)	0.056
	Male	16	(44)	15	(42)	

Residency	Non-northern	21	(58)	23	(64)	0.114
	Northern	15	(42)	13	(36)	

Comorbidity	<1	‡	‡	‡	‡	0.244
	≥1	‡	‡	‡	‡	
BMI (kg/m^2^)		23.98 (3.31)		24.70 (3.02)		0.228

Social-economic status	Minimum wage or lower	9	(25)	7	(19)	0.134
	Higher	27	(75)	29	(81)	

Smoking	No	24	(67)	26	(72)	0.121
	Yes	12	(33)	10	(28)	

Clinical T-stage	T1–T2	16	(44)	15	(42)	0.056
	T3–T4	20	(56)	21	(58)	

Clinical N-stage	N0–N2	7	(19)	7	(19)	0.056
	N3	29	(81)	29	(81)	

Tumor size (mm)		45.39 (18.90)		45.28 (22.82)		0.005

ECOG PS	0-1	‡	‡	‡	‡	0.109
	2	‡	‡	‡	‡	

BMI, body mass index; ECOG PS, eastern cooperative oncology group performance status; PS, propensity score; RT, radiotherapy; SD, standard deviation. ^†^Rounded. ^‡^The exact numbers are not reported because of a Health and Welfare Data Science Center (HWDC) database center policy to avoid numbers ≤2 in single cells.

**Table 3:** Characteristics of patients included in the second supplementary analysis.

		RT group (*n* = 35)		Non-RT group (*n* = 229)		Standardized difference^†^	
		Number or mean (SD)^†^	(%)^†^	Number or mean (SD)^†^	(%)^†^	Before PSW	After PSW
Age (years)		62.97 (8.58)		64.60 (8.30)		0.193	≈0

Sex	Female	20	(57)	158	(69)	0.247	≈0
	Male	15	(43)	71	(31)		

Residency	Non-northern	18	(51)	160	(70)	0.384	≈0
	Northern	17	(49)	69	(30)		

Comorbidity	<1	32	(91)	204	(89)	0.079	≈0
	≥1	3	(9)	25	(11)		

BMI (kg/m^2^)		25.23 (5.33)		24.01 (3.71)		0.266	≈0

Social-economic status	Minimum wage or lower	6	(17)	70	(31)	0.319	≈0
	Higher	29	(83)	159	(69)		

Smoking	No	25	(71)	178	(78)	0.145	≈0
	Yes	10	(29)	51	(22)		

Clinical T-stage	T1–T2	14	(40)	95	(41)	0.030	≈0
	T3–T4	21	(60)	134	(59)		

Clinical N-stage	N0–N2	9	(26)	49	(21)	0.030	≈0
	N3	26	(74)	180	(79)		

Tumor size (mm)		45.46 (19.12)		42.97 (20.74)		0.125	≈0

ECOG PS	0-1	31	(89)	212	(93)	0.137	≈0
	2	4	(11)	17	(7)		

BMI, body mass index; ECOG PS, eastern cooperative oncology group performance status; PS, propensity score; RT, radiotherapy; SD, standard deviation. ^†^Rounded.

**Table 4:** Characteristics of patients included in the third supplementary analysis.

		RT group (*n* = 8)		Non-RT group (*n* = 42)		Standardized difference^†^	
		Number or mean (SD)^†^	(%)^†^	Number or mean (SD)^†^	(%)^†^	Before PSW	After PSW
Age (years)		69.13 (6.66)		65.21 (5.83)		0.624	≈0

Sex	Female	4	(50)	31	(74)	0.506	≈0
	Male	4	(50)	11	(26)		

Residency	Non-northern	5	(62)	33	(79)	0.358	≈0
	Northern	3	(38)	9	(21)		

Comorbidity	<1	‡	‡	‡	‡	0.501	≈0
	≥1	‡	‡	‡	‡		

BMI (kg/m^2^)		23.53 (3.15)		24.35 (4.33)		0.217	≈0

Social-economic status	Minimum wage or lower	‡	‡	‡	‡	0.285	≈0
	Higher	‡	‡	‡	‡		

Smoking	No	‡	‡	‡	‡	0.144	≈0
	Yes	‡	‡	‡	‡		

Clinical T-stage	T1–T2	‡	‡	‡	‡	0.133	≈0
	T3–T4	‡	‡	‡	‡		

Clinical N-stage	N0–N2	4	(50)	13	(31)	0.133	≈0
	N3	4	(50)	29	(69)		

Tumor size (mm)		52.38 (22.93)		42.71 (19.87)		0.450	≈0

ECOG PS	0-1	‡	‡	‡	‡	0.501	≈0
	2	‡	‡	‡	‡		

BMI, body mass index; ECOG PS, eastern cooperative oncology group performance status; PS, propensity score; RT, radiotherapy; SD, standard deviation. ^†^Rounded. ^‡^The exact numbers are not reported because of a health and welfare data science center (HWDC) database center policy to avoid numbers ≤2 in single cells.

## Data Availability

The data that support the findings of this study are available upon reasonable request and with permission of Health and Welfare Data Science Center, Ministry of Health and Welfare, Executive Yuan, Taiwan, but restrictions apply to the availability of these data, which were used under license for the current study and so are not publicly available.

## Additional Points

*Key Points*. Significant findings of the study: our study suggests that the addition of radiotherapy to first- or second-generation EGFR TKI treatment did not significantly impact the overall survival of patients with clinical stage IIIB or IIIC lung adenocarcinoma and EGFR mutations. What this study adds? To our knowledge, this nonrandomized study using real-world data provides the first population-based investigation of this topic.

## Supplementary Materials

Supplementary Materials Supplementary material Table S1: characteristics of patients included in the fourth supplementary analysis.
