# Supplementary material for: Efficacy of Definitive Radiotherapy for Patients with Clinical Stage IIIB or IIIC Lung Adenocarcinoma and Epidermal Growth Factor Receptor (EGFR) Mutations Treated Using First- or Second-Generation EGFR Tyrosine Kinase Inhibitors
Source: Can Respir J. 2024 Mar 5;2024:8889536. doi: 10.1155/2024/8889536 (PMC10932622; doi:10.1155/2024/8889536)
Supplement: Supplementary Materials — Supplementary material Table S1: characteristics of patients included in the fourth supplementary analysis. [file 8889536.f1.docx]

Supplementary material table S1. Characteristics of patients included in the fourth supplementary analysis.

|  |  | RT group (n = 28) | | Non-RT group (n = 229) | | Standardized difference^†^ | |
| --- | --- | --- | --- | --- | --- | --- | --- |
|  |  | Number or mean (SD)^†^ | (%)^†^ | Number or mean (SD)^†^ | (%)^†^ | Before  PSW | After  PSW |
| Age (years) |  | 63.25 (8.58) |  | 64.60 (8.30) |  | 0.160 | ≈ 0 |
| Sex | Female | 20 | (56) | 158 | (69) | 0.280 | ≈ 0 |
|  | Male | 16 | (44) | 71 | (31) |  |  |
| Residency | Non-northern | 17 | (47) | 160 | (70) | 0.472 | ≈ 0 |
|  | Northern | 19 | (53) | 69 | (30) |  |  |
| Comorbidity | <1 | 33 | (92) | 204 | (89) | 0.088 | ≈ 0 |
|  | ≥1 | 3 | (8) | 25 | (11) |  |  |
| BMI (kg/m^2^) |  | 24.76 (4.40) |  | 24.01 (3.71) |  | 0.186 | ≈ 0 |
| Social-economic status | Minimum wage  or lower | 8 | (22) | 70 | (31) | 0.190 | ≈ 0 |
|  | Higher | 28 | (78) | 159 | (69) |  |  |
| Smoking | No | 25 | (69) | 178 | (78) | 0.189 | ≈ 0 |
|  | Yes | 11 | (31) | 51 | (22) |  |  |
| Clinical T-stage | T1–T2 | 16 | (44) | 95 | (41) | 0.060 | ≈ 0 |
|  | T3–T4 | 20 | (56) | 134 | (59) |  |  |
| Clinical N-stage | N0–N2 | 10 | (28) | 49 | (21) | 0.060 | ≈ 0 |
|  | N3 | 26 | (72) | 180 | (79) |  |  |
| Tumor size (mm) |  | 44.61 (19.87) |  | 42.97 (20.74) |  | 0.081 | ≈ 0 |
| ECOG PS | 0–1 | 32 | (89) | 212 | (93) | 0.127 | ≈ 0 |
|  | 2 | 4 | (11) | 17 | (7) |  |  |

BMI, body mass index; ECOG PS, Eastern Cooperative Oncology Group performance status; PSW, propensity score weighting; RT, radiotherapy; SD, standard deviation.

^†^Rounded.
